# Tunable degrees of neurodegeneration in rats based on microsphere-induced models of chronic glaucoma

**DOI:** 10.1038/s41598-022-24954-4

**Published:** 2022-11-30

**Authors:** María Jesús Rodrigo, Irene Bravo-Osuna, Manuel Subias, Alberto Montolío, José Cegoñino, Teresa Martinez-Rincón, Silvia Mendez-Martinez, Alba Aragón-Navas, David Garcia-Herranz, Luis Emilio Pablo, Rocío Herrero-Vanrell, Amaya Pérez del Palomar, Elena Garcia-Martin

**Affiliations:** 1 Department of Ophthalmology, Miguel Servet University Hospital, Zaragoza, Spain; 2 Innovation, Therapy and Pharmaceutical Development in Ophthalmology (InnOftal) Research Group UCM 920415, Department of Pharmaceutics and Food Technology, Faculty of Pharmacy, Complutense University of Madrid, Madrid, Spain; 3 Thematic Research Network in Ophthalmology (Oftared), Carlos III National Institute of Health, Madrid, Spain; 4 Biomaterials Group, Aragon Engineering Research Institute (I3a), University of Zaragoza, Zaragoza, Spain; 5 Department of Mechanical Engineering, University of Zaragoza, Zaragoza, Spain; 6 Miguel Servet Ophthalmology Research Group (GIMSO), Aragon Health Research Institute (IIS Aragón), University of Zaragoza, Zaragoza, Spain; 7 Health Research Institute of the San Carlos Clinical Hospital (IdISSC), Madrid, Spain; 8 C/Padre Arrupe, Servicio de Oftalmología, Edificio de Consultas Externas, Planta 1, 50009 Zaragoza, Spain

**Keywords:** Biotechnology, Drug discovery, Neuroscience

## Abstract

This study compares four different animal models of chronic glaucoma against normal aging over 6 months. Chronic glaucoma was induced in 138 Long–Evans rats and compared against 43 aged-matched healthy rats. Twenty-five rats received episcleral vein sclerosis injections (EPIm cohort) while the rest were injected in the eye anterior chamber with a suspension of biodegradable microspheres: 25 rats received non-loaded microspheres (N-L Ms cohort), 45 rats received microspheres loaded with dexamethasone (MsDexa cohort), and 43 rats received microspheres co-loaded with dexamethasone and fibronectin (MsDexaFibro cohort). Intraocular pressure, neuroretinal function, structure and vitreous interface were evaluated. Each model caused different trends in intraocular pressure, produced specific retinal damage and vitreous signals. The steepest and strongest increase in intraocular pressure was seen in the EPIm cohort and microspheres models were more progressive. The EPIm cohort presented the highest vitreous intensity and percentage loss in the ganglion cell layer, the MsDexa cohort presented the greatest loss in the retinal nerve fiber layer, and the MsDexaFibro cohort presented the greatest loss in total retinal thickness. Function decreased differently among cohorts. Using biodegradable microspheres models it is possible to generate tuned neurodegeneration. These results support the multifactorial nature of glaucoma based on several noxa.

## Introduction

Glaucoma is a multifactorial and degenerative disease of the retina and optic nerve that causes progressive and irreversible blindness. It is estimated that this disease will affect over 100 million people worldwide by 2040^1^. The main modifiable risk factor for glaucoma development and progression is elevated intraocular pressure, which causes primary retinal ganglion cell degeneration by axonal compression in the lamina cribosa^2^. This damage then spreads via anterograde and retrograde neurodegenerative pathways^3^. Since glaucomatous damage continuesto proliferate in some patients despite appropriate intraocular pressure management, other factors such as fluctuation^4^ or immunity^5–7^ have been suggested as influencing the pathogenesis of secondary degeneration. In secondary degeneration, creation of an excitotoxic environment due to glutamate, ionic imbalance or free radicals increases damage to neighboring neurons, leading to apoptosis and necrosis of surrounding cells^8^.

In recent decades, to study anterograde neurodegeneration in the retina and optic nerve several animal models were created by increasing intraocular pressure using pre-post-trabecular methods^9^. Animal studies, however, produced differing neurodegenerative findings, possibly due to differences in methodological design or the different animals and ocular hypertensive models used. It is important to note that in animal research neuroretinal damage usually occurred in early phases and that, therefore, the follow-up times evaluated were short. These premises differ widely with the most characteristic feature of primary open-angle glaucoma, namely chronicity^10^. Microbead models offer advantages such as minimally invasive injections^11^ and modulated intraocular pressure achieved by managing the timing of injections^12^. Recent developments in drug delivery systems such as poly(lactic-co-glycolic acid) (PLGA) microspheres have not only proven their usefulness for treating posterior pole pathologies^13^ but also for inducing chronic glaucoma models. Their beneficial properties, such as biodegradability and maintenance of a healthy ocular surface and anterior segment structures, make it possible to test animals repeatedly over time using optical coherence tomography (OCT) and/or electroretinography (ERG). In chronic glaucoma models produced by intracameral injection of PLGA microspheres, the progressive alteration of the trabecular meshwork by mechanical clogging was combined with pharmacological action via the different effects of the sustained release of the encapsulated active agent(s), permitting a decreasing number of inducing injections^14–16^. Computational and imaging technologies were used to analyze the neurodegenerative process, perform follow-up and assess the efficacy of neuroprotective treatments^17–22^ on the retina, optic nerve and vitreous. Histological studies correlated with OCT findings^22–24^. In general terms, although previous studies presented the gross effect of neurodegeneration/protection on the neuroretina, characterizing the damage to retinal structures over time was challenging, possibly due to difficulties in carrying out longer longitudinal prospective studies because of the lack of chronic glaucoma models with increased and sustained intraocular pressure.

A long-term goal of glaucoma treatment is to personalize therapy^25^ according to the specific stage of the disease, not only treating the pathology when it is well established, but also adopting a preventive approach. Detailed knowledge of the processes involved in neuroretinal degeneration is needed to distinguish between the early phases of the disease and progression, and even to catalog it based on different noxa involved. The creation and comparison of several chronic glaucoma models is key to understanding the milestones marking the different stages and classes of glaucoma. This study compares four different chronic glaucoma models with healthy control rats via functional and structural examinations of intraocular pressure, OCT and ERG data over 6 months’ follow-up. In the chronic glaucoma models, ocular hypertension was increased at different points in time by injecting non-loaded microspheres and microspheres loaded with active agents such as dexamethasone and fibronectin to produce different and specific damage.

## Methods, intervention, or testing

### Chemicals and reagents

Poly (D, L-lactide-co-glycolide) (PLGA) 50:50 (12,000 g/mol) was purchased from Evonik Industries (Essen, Germany). Dexamethasone was supplied by Sigma-Aldrich (St. Louis Mo., USA) (purity > 98%). Fibronectin, fibronectin ELISA and reactants (Reagent Diluent DY995, Wash Buffer WA126, Stop Solution DY994 and Substrate Reagent Pack DY999) were purchased from R&D Systems (Minneapolis, MN, USA). Polyvinyl alcohol (PVA, 67,000 g/mol) was obtained from Merck KGaA (Darmstadt, Germany), and methylene chloride was obtained from PanReac AppliChem (Barcelona, Spain).

### Methods

#### Microsphere fabrication

PLGA microspheres were fabricated by solvent extraction–evaporation to an oil-in-water (O/W) emulsion or to a water-in-oil-in-water emulsion depending on the solubility of the active agents included in the formulation^14–16^.

Briefly, the organic phases were prepared by dissolving the polymer in methylene chloride (20% w/v). For the formulations containing dexamethasone, 40 mg of the corticoid were dispersed in the polymeric solution (Ultrasons, J.P. Selecta, Barcelona, Spain). In the case of the formulation containing fibronectin, 20 µL of the protein solution in aqueous media phase (0.214% w/v) were emulsified with the polymeric solution containing dexamethasone (Sonicator XL, Heat Systems, Inc., Farmingdale, NY, USA) for 30 s at 4 °C to create the initial W_1_/O emulsion. In parallel, the external aqueous phase was prepared by dissolving PVA in MilliQ water (1% w/v).

The complete organic phase and the external aqueous phases were subsequently emulsified (PolytronVR RECO, Kinematica, GmbHT PT3000, Lucerne, Switzerland) at 7000 rpm for 1 min. The resulting O/W or W/O/W emulsions were maintained for 3 h under stirring at room temperature in the maturation phase composed of 100 mL of PVA MilliQ water solution (0.1% w/v). Finally, the microspheres were washed with MilliQ in order to remove the surfactant and were sieved to obtain the 20–10 mm fraction. Afterwards, they were freeze-dried (freezing: 60 °C/15 min.; drying: 60 °C/12 h/0.1 mBar) and stored at 30 °C in dry conditions until use.

#### Microsphere characterization

The different types of fabricated microsphere were characterized in terms of production percentage of the desired size fraction (20–10 µm), considering the initial amounts of the different components used to prepare the formulations and the final amount of microspheres obtained. Average particle size and particle size distribution were measured by dual light scattering (Microtrac S3500 Series Particle Size Analyzer, Montgomeryville, PA, USA) and their external morphology was evaluated by scanning electron microscopy (Jeol, JSM-6335F, Tokyo, Japan).

The microspheres loaded with different active agents were also evaluated with regard to encapsulation efficacy and in vitro release profile, both for dexamethasone and fibronectin. To evaluate the dexamethasone encapsulation efficacy, 1 mg of microspheres was dissolved in 2.5 mL of dichloromethane. Subsequently, 6 mL of methanol were added in order to precipitate the polymer and collect the corticoid dissolved in the dichloromethane:methanol mixture after centrifugation (5,000 rpm, 5 min, 20 °C) and filtration (0.22 µm). Unfortunately, the lability of the protein made liquid–liquid extraction (as can be done with other proteins) impossible^26^. To carry out the in vitro release studies, 2.5 mg of microspheres were suspended in 1 mL of release medium (PBS isotonized with NaCl pH 7.4, including sodium azide 0.02% w/v) and subjected to gentle agitation and physiological temperature (100 rpm, 37 °C, Memmert Shaking Bath, Memmert, Schwabach, Germany). At predetermined times, after gentle centrifugation (5,000 rpm for 5 min, 20 °C) the entire liquid medium was extracted, filtered (0.22 μm) and used for subsequent quantification of the released active agents. The remaining microspheres were suspended in fresh release medium until the next sample was taken, repeating the entire process. As in the ocular hypertension animal model created with microspheres loaded with dexamethasone, a second dose (same amount of microspheres) was reinjected 4 weeks after the initial one. In the case of the in vitro release tests, this reinjection was simulated by adding a second 2.5 mg dose of microspheres to the release study at 28 days. The in vitro release assays were performed in *sink* conditions.

The quantification methods for both compounds—HPLC/MS system consisting of a liquid chromatography instrument (Waters 1525 binary HPLC pump and Waters 2707 autosampler connected to a MS detector; Waters 3100 single quadrupole mass spectrometer) for dexamethasone, and ELISA (DuoSet Human Fibronectin DY1918-05, R&D Systems, Minneapolis, MN, USA) for the protein—were duly set up and validated. Detailed characterization of the different microspheres used in these studies was compiled from various previous publications^14–16^.

##### Animal welfare

The procedure with animals was carried out at the Biomedical Research Center of Aragon. The experiment was approved by the Ethics Committee for Animal Research (PI34/17). All experiences were performed in accordance with the Association for Research in Vision and Ophthalmology Statement for the Use of Animals and in accordance with the Animal Research: Reporting of In Vivo Experiments guideline. The experiments were carried in accordance with the relevant guidelines and regulations. The study is reported in accordance with ARRIVE (Animal Research: Reporting of In Vivo Experiments) guidelines. The animals were housed in standard cages with environmental enrichment and water and food ad libitum in 12 h dark–light cycled rooms under controlled temperature (22 °C) and relative humidity (55%) conditions. All animal procedures were performed at controlled temperature using warm pads and the rats were left to recover in an oxygen-enriched (2.5%) atmosphere.

##### Animal cohorts

One hundred and eighty-one Long–Evans rats (40% males, 60% females; 4 weeks old; initial weights ranging from 50–100 g) were used. The animals were divided in 5 different cohorts: healthy control cohort (43 animals that did not receive ocular hypertensive injections), EPIm cohort (25 animals with sclerosis of the episcleral veins^27^), N-L Ms cohort (25 animals with a 2 μL suspension of biodegradable non-loaded PLGA microspheres^14^ injected into the anterior chamber of the eye), MsDexa cohort (43 animals injected with a 2 μL suspension of biodegradable PLGA microspheres loaded with Dexamethasone^15^), and MsDexaFibro cohort (45 animals injected with a 2 μL suspension of biodegradable PLGA microspheres loaded with dexamethasone and fibronectin)^16^.

##### Anesthesia, euthanasia and other drugs

Intraocular pressure measurements and ocular injections were performed under gas anesthesia with a mixture of 3% sevoflurane gas and 1.5% oxygen. To prevent infection and/or discomfort caused by the ocular injections, subcutaneous analgesia (dilution 1/10 of buprenorphine [0.05 mg/kg]), topical anesthesia (tetracaine 1 mg/ml + oxybuprocaine 4 mg/ml), antiseptic eye drops (iodopovidone 0.1%) and eye lubrication (hypromellose 2%) were used. Electroretinogram and OCT readings were taken under anesthesia administered by intraperitoneal injection (60 mg/kg of ketamine + 0.25 mg/kg of dexmedetomidine). Anesthetic (previously mentioned) and eye drops for pupillary dilation (tropicamide 10 mg/ml and phenylephrine 100 mg/ml) were also administered. For euthanasia*,* the animals were sedated (with the previous intraperitoneal injection anesthesia) and euthanized under humane conditions using an intracardiac injection of sodium thiopental (25 mg/ml).

##### Ocular hypertensive injections and ophthalmological studies

**Figure 1:**
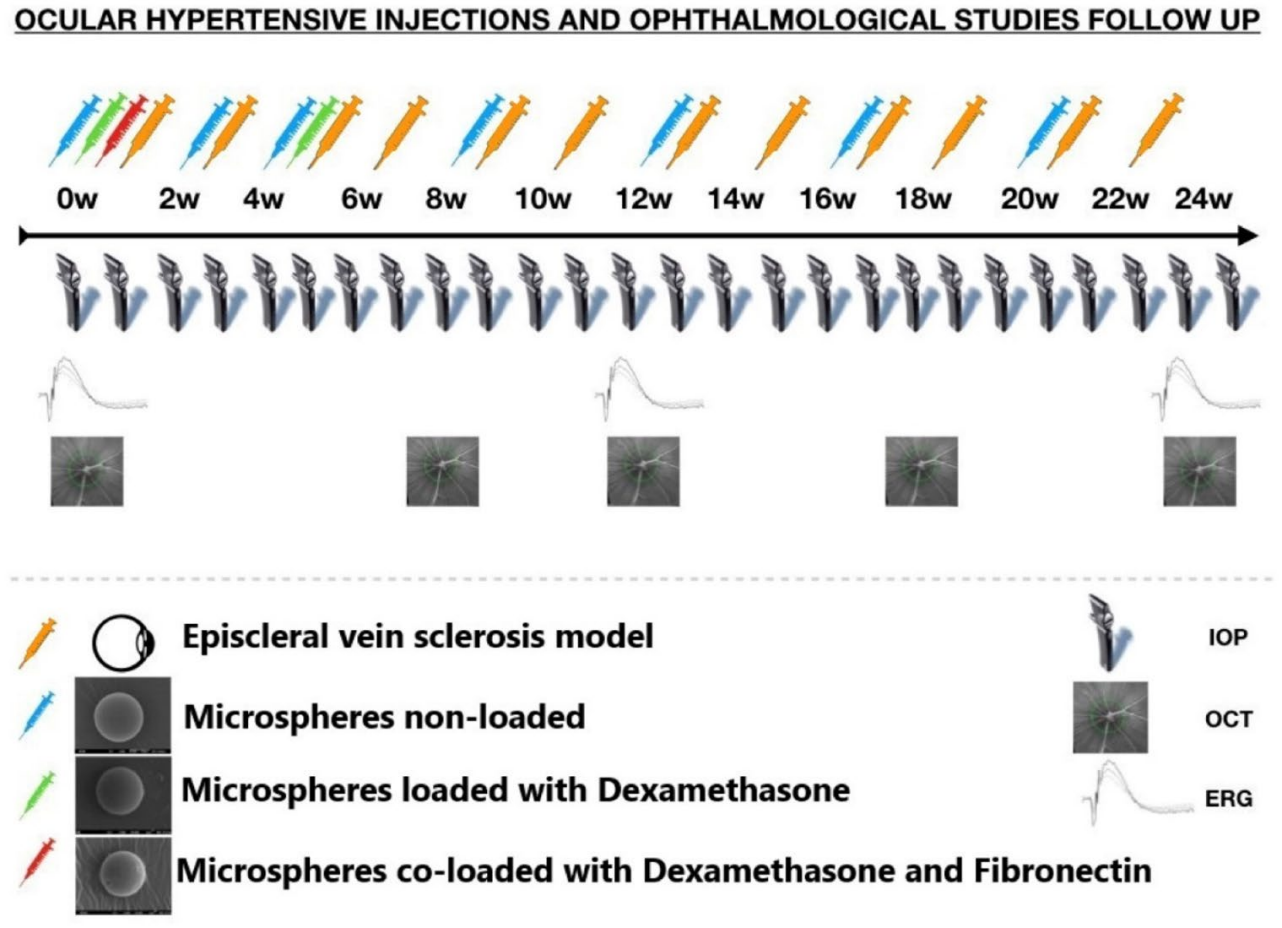
Ocular hypertensive injections and ophthalmological studies. *IOP* intraocular pressure; *OCT* optical coherence tomography; *ERG* electroretinography; *w* week. The microspheres external morphology images were obtained by scanning electron microscopy (Jeol, JSM-6335F, Tokyo, Japan). The rat optic nerve head images were adquired by Spectralis optical coherence tomography device (Heidelberg Engineering, Germany).

Figure 1 shows the frequency of ocular injections in each cohort and ophthalmological study. All animals (except control cohort) were injected in the right eye by the same researcher under aseptic conditions.

*Intraocular pressure* was measured using the Tonolab rebound tonometer (Tonolab; Tiolat Oy Helsinki, Finland). All measurements were recorded every week in the morning in both eyes (right eye first) in all remaining animals of each time explored (total—6 animals at each time explored). As recommended, each animal was measured in under 3 minutes^28^ to avoid the hypopressure effect due to anesthesia. The intraocular pressure value was averaged from 18 consecutive measurements.

*Optical coherence tomography* The neuroretinal structures and vitreous interface were analyzed using OCT technology (Spectralis OCT, Heidelberg Engineering, Germany). For each OCT experiment, at least 6 animals (both sexes) were used at each time explored. The times explored were 0, 2, 4, 6, 8, 12, 18 and 24 weeks. The following three protocols (Retinal Posterior Pole [RPP], Retinal Nerve Fiber Layer [RNFL] and Ganglion Cell Layer [GCL]) were performed using automatic segmentation, eye-tracking, follow-up application, and a contact lens adapted on the rat cornea to acquire higher quality images. These protocols analyzed an area centered on the optic disc (instead of the fovea, which rats do not have), using 61 b-scans. The RPP and GCL protocols analyzed the Early Treatment Diabetic Retinopathy Study areas^29^, compound by one central 1 mm circle, four inner (inferior, superior, nasal, temporal) and four outer (inferior, superior, nasal, temporal) sectors from rings measuring 2 and 3 mm in diameter, respectively. The total volume was also registered. The RNFL protocol studied the sectors (inferotemporal, inferonasal, superotemporal, superonasal, nasal, and temporal). The limits of the retinal thickness using this software are the retinal pigment epithelium and the inner limiting membrane; the limits of the RNFL are the GCL boundaries and the inner limiting membrane; and the limits of the GCL are the inner nuclear layer boundaries and the RNFL.

To study vitreous parainflammation, the vitreous interface was scanned using the RPP protocol. The OCT images were exported in Audio Video Interleaveformat. Each video was composed of 61 cross-sectional b-scans with a total of 1536 × 496 pixels each. These videos were analyzed using a custom program implemented in Matlab (version R218a, Mathworks Inc., Natick, MA, USA). The imaging data were analyzed by a masked reader. Vitreous/retinal pigment epithelium relative intensity was quantified as an indirect measure of immune response, and vitreous opacities, closely related to the immune cells, were analyzed as in our previous paper^22^. A total of 225 right eye OCT videos were analyzed (72 from the EPIm cohort, 38 from N-L Ms, 40 from MsDexa, 44 from MsDexaFibro and 31 from the control cohort).

*Electroretinography* Neuroretinal function was obtained using the full-field scotopic ERG and Photopic Negative Response (PhNR) protocols (Roland consult, RETIanimal ERG, Germany). For each ERG experiment, at least 6 animals (both sexes) were used at each time explored. The times explored were 0, 12 and 24 weeks. For scotopic ERG test, the animals were dark-adapted for 12 h and for the PhNR protocol light adaptation to a blue background, and red Light Emitting Diode flash was used as stimulus. Both eyes’ pupils were fully dilated with mydriatic eye drops for bilateral simultaneous testing^30^. Electrode impedance discrepancy < 2 kΩ between electrodes was accepted. A multistep procedure^31^ comprising 7 steps of increasing luminance intensity and different intervals were used, which registered rod response (steps 1 to 5), mixed rod-cone response (step 6) and oscillatory potentials (step 7), as described in^14,15^*.* Latency (in milliseconds) and amplitude (in microvolts) were studied in a, b and PhNR waves.

### Statistical analysis

The data were recorded in an Excel database and statistical analysis was performed using Statistical Package for Social Sciences 20.0 version (SPSS Inc., Chicago, IL). The Kolmogorov–Smirnov test was used to evaluate sample distribution. The differences between the five cohorts were evaluated using the Analysis Of Variance (ANOVA) test and the *post-hoc* Scheffé test. All values were expressed as mean ± standard deviations. Values of *p* < 0.05 (expressed as *) were considered to be statistical significance. To avoid a high false-positive rate, the Bonferroni correction for multiple comparisons was applied and the level of significance for each variable was established based on Bonferroni calculations.

## Results

As shown in Table 1, the microsphere formulations used to create the different animal models had a particle size in the desired range (20–10 µm), a homogeneous particle size distribution, a spherical and defined shape and an acceptable production yield in all cases. Evaluation of the external morphology based on scanning electron microscopy studies showed that the dexamethasone-loaded and non-loaded microspheres, for which the fabrication process was based on formation of a simple emulsion, presented pore-free surfaces. However, in the case of the microspheres loaded with fibronectin and dexamethasone, small pores were observed as a result of the double emulsion technique used in their manufacture. The dexamethasone load was optimal in the two formulations that contain this active agent, it being slightly higher in the formulation that also contains fibronectin. As commented in the Materials and Methods section, due to the high lability of the protein it was not possible to extract it directly from the microspheres. Therefore, the cumulative value obtained at the end of the microsphere in vitro release assay was taken as the microspheres’ fibronectin load value. A more detailed description of these results can be obtained from previous published studies^14–16^.

**Figure 2:**
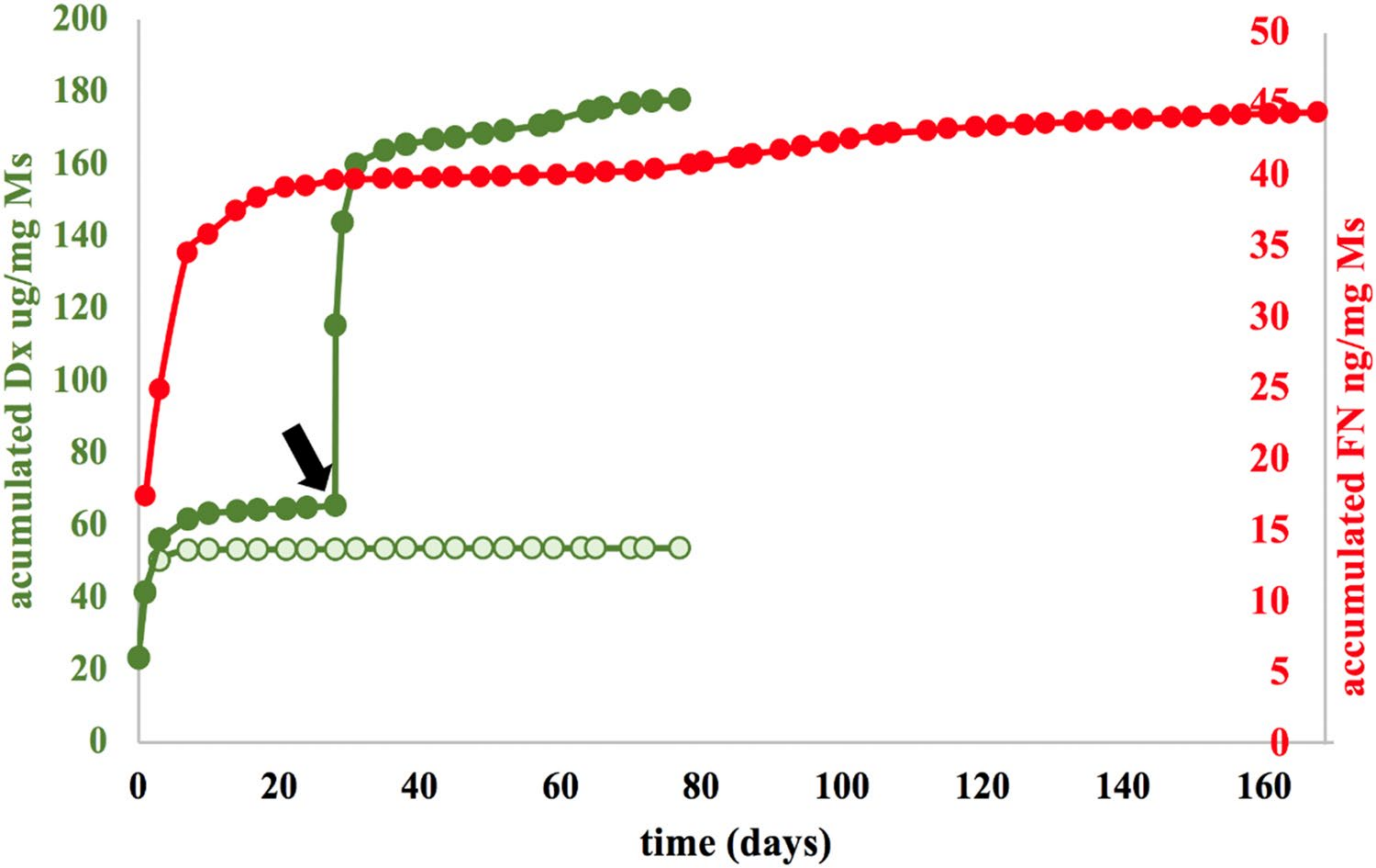
Cumulative in vitro release of dexamethasone (μg/mg Ms) [in green] from Dexamethasone-loaded microspheres [dark green] and Dexamethasone/Fibronectin-loaded microspheres [light green]. Cumulative in vitro release of fibronectin (ng/mg Ms) [in red]. Release media: PBS (pH 7.4 isotonized with NaCl) and 0.02% Na azide. *Note*: To simulate the in vivo conditions of the animal model obtained by two injections of Dexamethasone-loaded microspheres, a second dose of microspheres was included in the release media at 28 days [black arrow]. *DX* dexamethasone, *FN* fibronectin, *Ms* microspheres.

As can be seen in Fig. 2, the PLGA microparticulate systems created progressively released their content for several weeks. The in vitro dexamethasone release assay that simulated the two injections needed to create the animal model of glaucoma using MsDexa lasted about 80 days. In vitro dexamethasone release values were also obtained up to that point in time when the corticoid was co-microencapsulated and subsequently co-released with fibronectin from MsDexaFibro, this time without simulating any re-injection. For its part, the protein, after initial rapid release, was released slowly through to the end of the assay (168 days).

**Table 1:** Physical and chemical characterization of the microsphere formulations used to create the different ocular hypertension animal models. Production yield (%); Mean particle size and particle size distribution, external morphology (*SEM*: scanning electron microscopy; Jeol, JSM-6335F, Tokyo, Japan), dexamethasone loading (µg DX/mg Ms) according to direct quantification after extraction from the microspheres (Ms), and fibronectin loading (ng FN/mg Ms) indirectly obtained as the final point of cumulative release in the in vitro release assay.

	N-L Ms	MsDexa	MsDexaFibro
Production yield (%)	39.37	77.34	55.14
Mean particle size (μm)	14.07 ± 1.07	13.13 ± 0.60	14.81 ± 0.30
Particle size distribution	monodisperse	monodisperse	monodisperse
External morphology (SEM)	Non-porous & smooth surface	Non-porous & smooth surface	Slightly porous & smooth surface
Dx loading (µg DX/mg Ms)	–	60.70 ± 1.03	71.94 ± 2.40
FN loading (ng DX/mg Ms)	–	–	74.47 ± 0.06

### Intraocular pressure analysis

**Figure 3:**
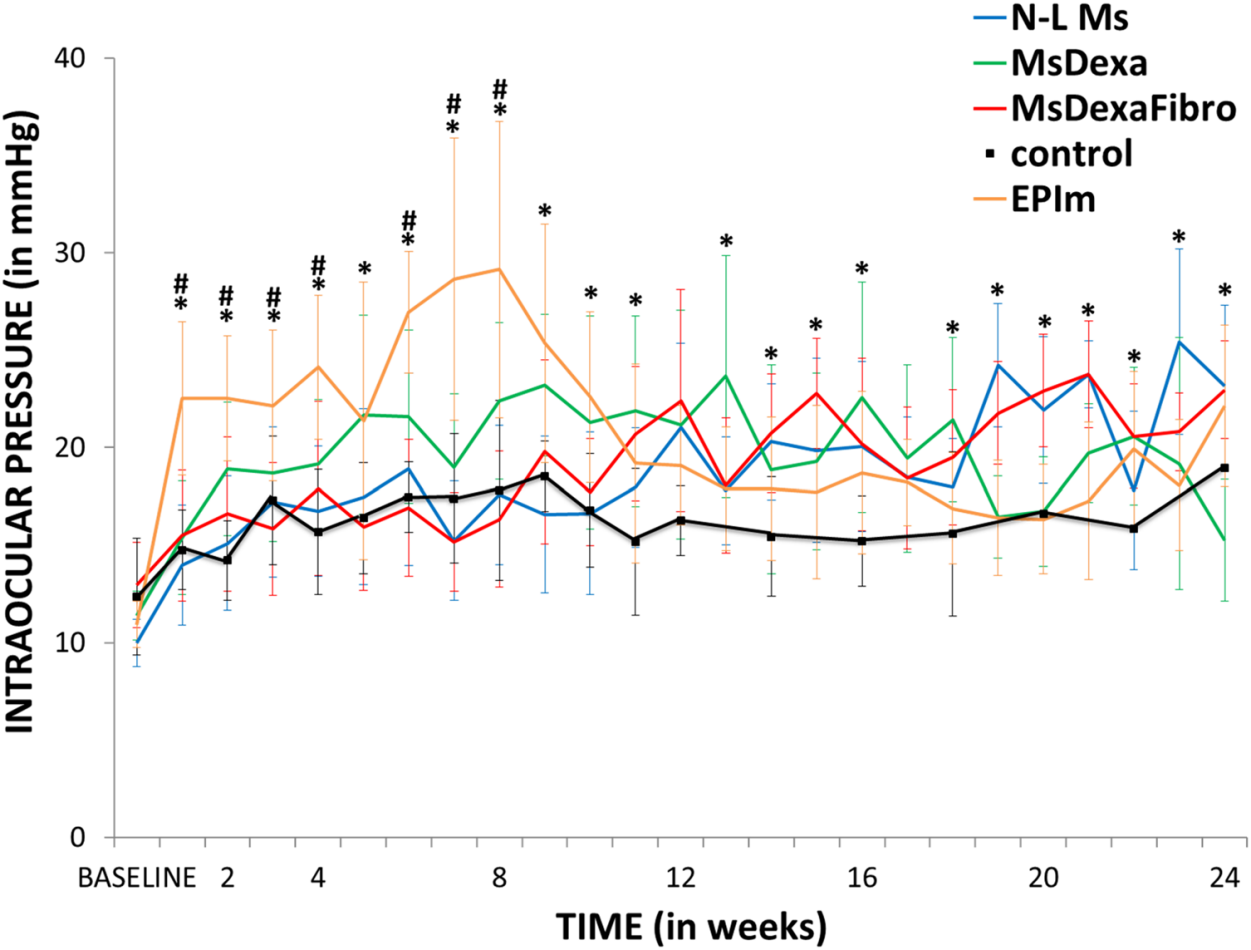
Comparison of intraocular pressure curves in different chronic glaucoma models. *N-L Ms*: cohort with non-loaded microspheres; *MsDexa*: cohort with microspheres loaded with dexamethasone; *MsDexaFibro*: cohort with microspheres loaded with dexamethasone and fibronectin; *EPIm*: cohort with sclerosis of the episcleral veins. *: statistical significance (*p* < 0.05) between glaucoma models and healthy controls (ANOVA); #: statistical significance between the episcleral vein sclerosis model and each microsphere model by Scheffé test. Note that at later times, differences were found between EPIm and some of the microsphere models, (data not shown). Differences were only included when found between the EPIm model and each of the microsphere models. As can be seen, this occurred only at earlier times.

All the induced chronic glaucoma models presented a progressive increase in intraocular pressure over the course of the study. The highest levels were detected in the EPIm cohort, especially in the early phases. Among the microspheres models, the MsDexa cohort presented the highest intraocular pressure levels and both the N-L Ms and MsDexaFibro cohorts behaved similarly, although the MsDexaFibro cohort presented the most evident fluctuations. The non-intervened healthy rats presented intraocular pressure values < 20 mmHg up to week 24 (Fig. 3). The percentage of ocular hypertensive eyes (with intraocular pressure > 20 mmHg) was analyzed. The EPIm cohort presented ocular hypertensive values in over 70% of eyes in the early phases (up to week 12), but these then decreased. Conversely, eyes in the N-L Ms and MsDexaFibro cohorts exhibited the opposite trend, with higher percentages of ocular hypertension in later phases (from week 12 through to the end). The MsDexa cohort presented the highest average ocular hypertension percentage of all cohorts over the course of the study. Analyzing ocular hypertension intensity by model, ocular hypertensive response was classified as low (increase of < 6 mmHg from baseline), medium (6–15 mmHg) or high (> 15 mmHg). A low–medium ocular hypertensive response was observed in all chronic glaucoma cohorts, with a low number (< 20%) of high responses. The EPIm cohort, however, presented a substantial high response in early phases.

### Optical coherence tomography analysis

**Figure 4:**
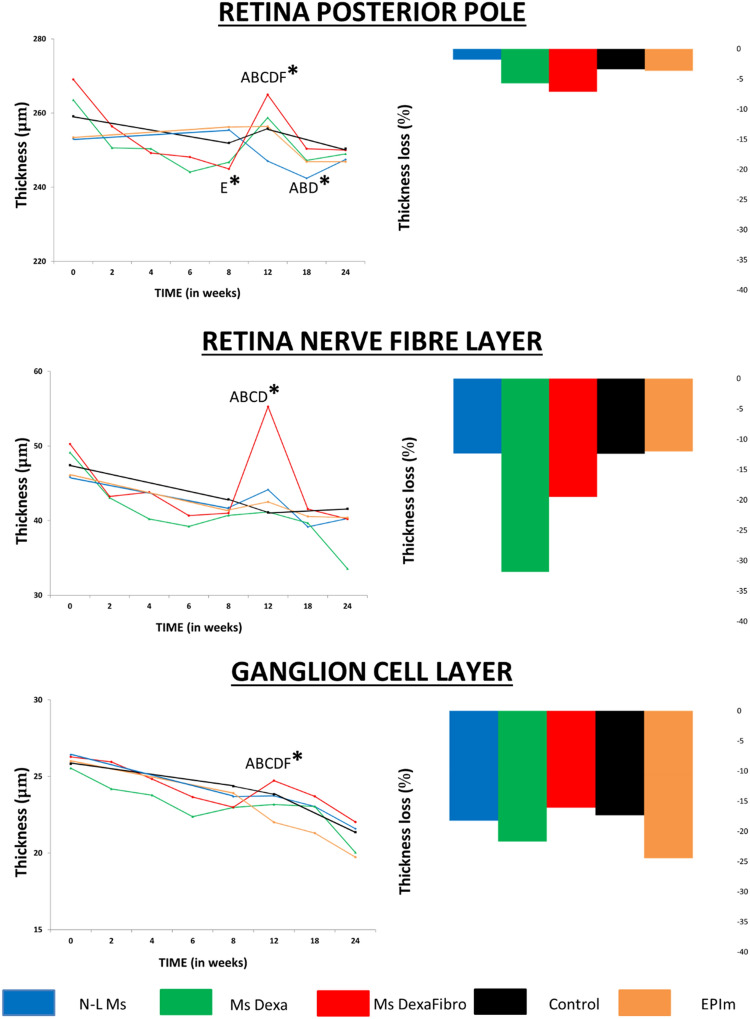
Comparison of structural neuroretinal studies using optical coherence tomography (OCT) between four chronic glaucoma models and healthy control cohorts Left: Neuroretinal thickness measured using OCT over 24 weeks. Right: Neuroretinal thickness loss percentage at the end of the study. *%*: percentage; *μm* thickness in microns; *N-L Ms* cohort with non-loaded microspheres; *MsDexa* cohort with microspheres loaded with dexamethasone; *MsDexaFibro* cohort with microspheres loaded with dexamethasone and fibronectin; *EPIm* cohort with sclerosis of the episcleral veins; *: statistical significance (*p* < 0.05) between glaucoma models and healthy controls (ANOVA); A: significant differences between N-L Ms and MsDexa; B: significant differences between MsDexa and MsDexaFibro; C: significant differences between MsDexa and controls; D: significant differences between MsDexa and EPIm; E: significant differences between MsDexaFibro and controls; F: significant differences between MsDexaFibro and EPIm; by Scheffé test.

The neuroretinal thickness of RPP, RNFL and GCL was quantified over the study and compared among the five cohorts. Although a progressive decrease in thickness was found, all cohorts suffered an increase in fluctuation in week 12. This was most intense in the MsDexaFibro cohort. The EPIm cohort presented the lowest thickness in RPP and GCL, and the MsDexa cohort presented the thinnest RNFL at the end of the study (Fig. 4 left). However, when calculating the percentage of neuroretinal loss from baseline, in the MsDexaFibro cohort the RPP presented the greatest loss. Comparisons between cohorts of the percentage loss at the end of the study (24 weeks) are shown in Fig. 4 (right).

**Figure 5:**
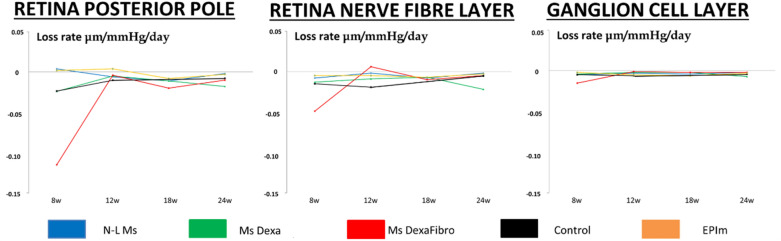
Standardization of neuroretinal thickness loss measured using optical coherence tomography between four chronic glaucoma models and healthy control cohorts. *w* week; *N-L Ms* cohort with non-loaded microspheres; *MsDexa* cohort with microspheres loaded with dexamethasone; *MsDexaFibro* cohort with microspheres loaded with dexamethasone and fibronectin; *EPIm* cohort with sclerosis of the episcleral veins.

The loss rate expressed in microns per mmHg and day extracted from averaging all OCT sectors was also quantified in right eyes and at various points in time in order to normalize the neuroretinal loss. All three explored parameters (RPP, RNFL and GCL) presented a negative loss rate over the course of the study. The loss rate behaved similarly in the GCL across all cohorts; it was lower in the RNFL in the chronic glaucoma cohorts than in the healthy control cohort; and it fluctuated in the RPP. Comparing all cohorts, the MsDexaFibro cohort presented the widest variations, especially in early phases (Fig. 5).

**Figure 6:**
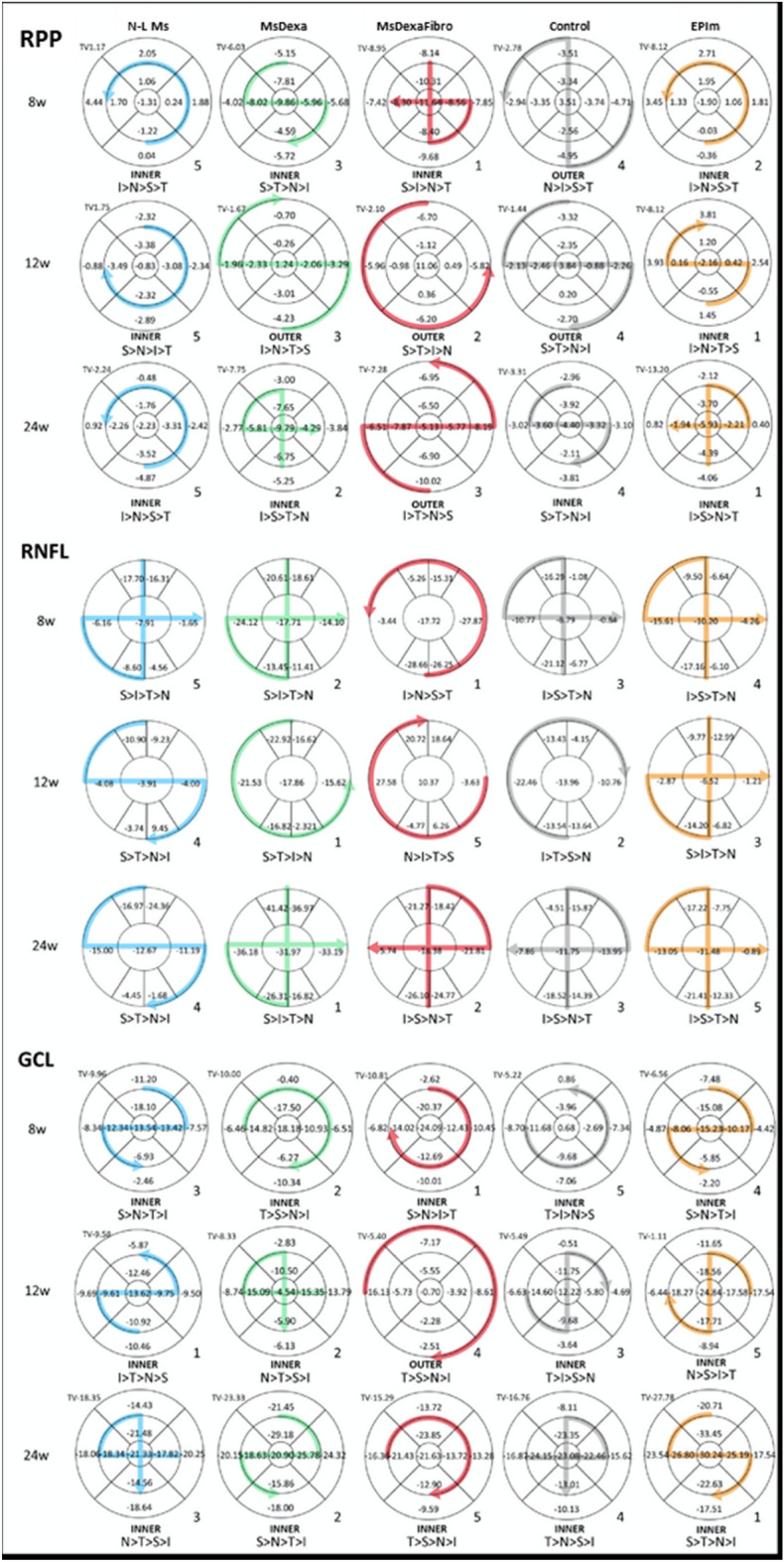
Percentage loss by optical coherence tomography sector and loss trend follow-up comparison among four chronic glaucoma models and healthy control cohorts. *w* week; *RPP* retina posterior pole; *RNFL* Retinal Nerve Fiber Layer; *GCL* Ganglion Cell Layer complex; *TV* total volume; > greater than; *S* superior; *I* inferior; *N* nasal; *T* temporal; *N-L Ms* cohort with non-loaded microspheres; *MsDexa* cohort with microspheres loaded with dexamethasone; *MsDexaFibro* cohort with microspheres loaded with dexamethasone and fibronectin; *EPIm* cohort with sclerosis of the episcleral veins. Grading: high (5) to low (1) based on total volume loss severity.

Percentage neuroretinal loss by OCT sector in the RPP, RNFL and GCL was quantified and loss tendency follow-up was analyzed to identify a topographical neurodegenerative pathway. Different patterns were found depending on the RPP, RNFL, and GCL parameter explored at the same time in the same cohort. Alternation in inner and outer sectors was observed in the RPP. In the GCL the inner sectors presented highest percentage loss, as did the vertical axis (superior and inferior sectors) in the RNFL. When comparing all the cohorts, similar patterns were found at the same explored week (week 8) in the RPP, RNFL and GCL, or at week 24 in the RNFL (Fig. 6).

**Figure 7:**
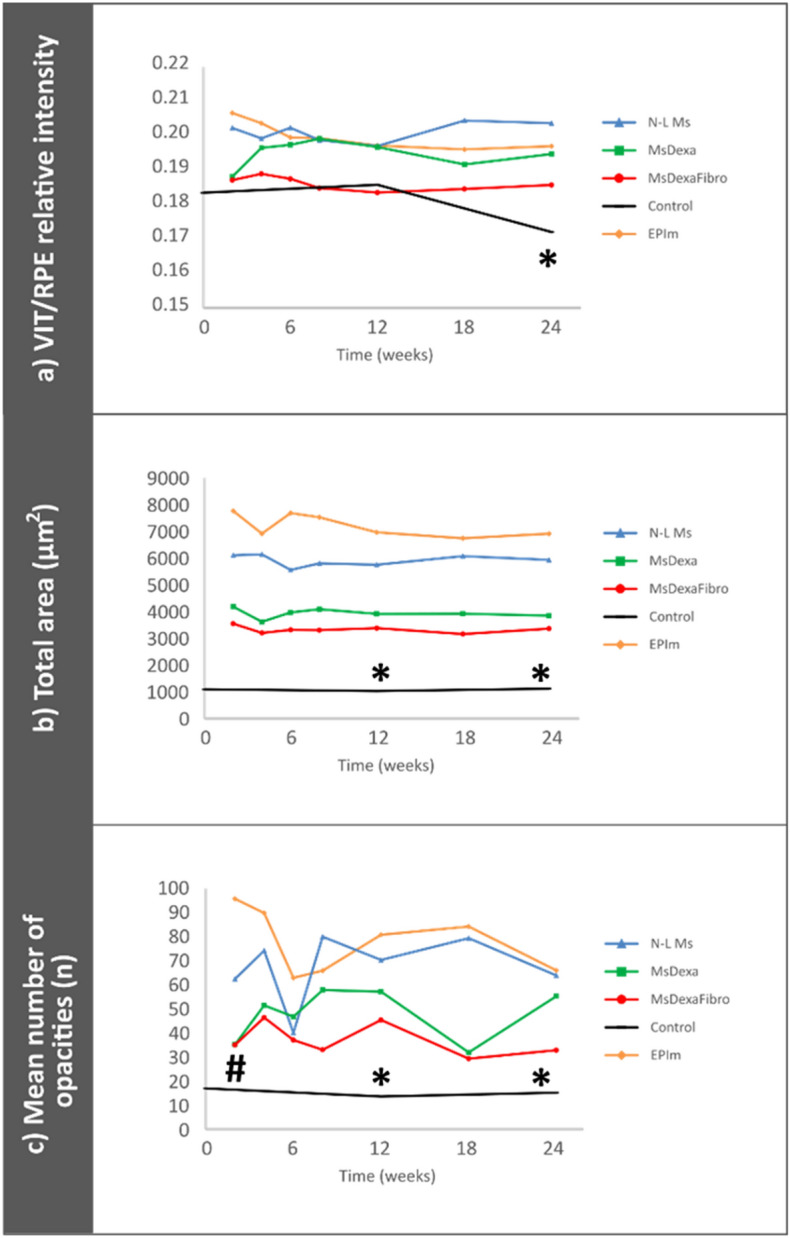
Vitreous parainflammation measured using optical coherence tomography. (**a**): Vitreous/retinal pigmented epithelium relative signal intensity; (**b**): total area of opacities to show changes in total immune response; (**c**): mean number of opacities to quantify immune cells. *N-L Ms* cohort with non-loaded microspheres; *MsDexa* cohort with microspheres loaded with dexamethasone; *MsDexaFibro* cohort with microspheres loaded with dexamethasone and fibronectin; *EPIm* cohort with sclerosis of the episcleral veins. *: statistical significance (*p* < 0.05) between glaucoma models and healthy controls (ANOVA); #: statistical significance between the episcleral vein sclerosis model and each microsphere model by Scheffé test. Note that at later times, differences were found between EPIm and some of the microsphere models, (data not shown). Differences were only included when found between the EPIm model and each of the microsphere models. As can be seen, this occurred only at earlier times.

Vitreous parainflammation was indirectly quantified by vitreous/retinal pigment epithelium relative intensity. OCT analysis of the vitreous detected higher intensities in all hypertensive models of chronic glaucoma versus healthy controls. After the first hypertensive injection, the EPIm model presented the highest intensity in the vitreous, followed by the anterior chamber microsphere injection-based models ordered by number of injections: N-L Ms (7 injections) > MsDexa with dexamethasone loading (2 injections) and MsDexaFibro with dexamethasone/fibronectin co-loading (1 injection). At the end of the study, the N-L Ms model (by multiple intraocular injection) outperformed EPIm (with more intervention but no ocular perforation) (Fig. 7a). In addition, at week 2, when all models had been injected only once, signal intensity in the steroid-induced models was similar in intensity to the healthy control cohort. Individual hyperreflective opacities were detected and the total area was quantified as a representation of overall immune response. The EPIm model presented the highest total area (approx. 8000 µm^2^), followed by N-L Ms (approx. 6000 µm^2^), MsDexa (approx. 4000 µm^2^) and MsDexaFibro (approx. 3200 µm^2^) versus the healthy control cohort (approx. 1,000 µm^2^), and all of them remained constant over time (Fig. 7b). When quantifying the mean number of opacities, EPIm also had a higher number of opacities (approx. 80): > N-L Ms (approx. 70) > MsDexa (approx. 45) > MsDexaFibro (approx. 35) > healthy control cohort (approx. 10). However, different patterns of fluctuation were observed in each glaucoma model. This contrasts with the healthy control cohort, which remained at consistently low levels (Fig. 7c).

### Electroretinography analysis

**Figure 8:**
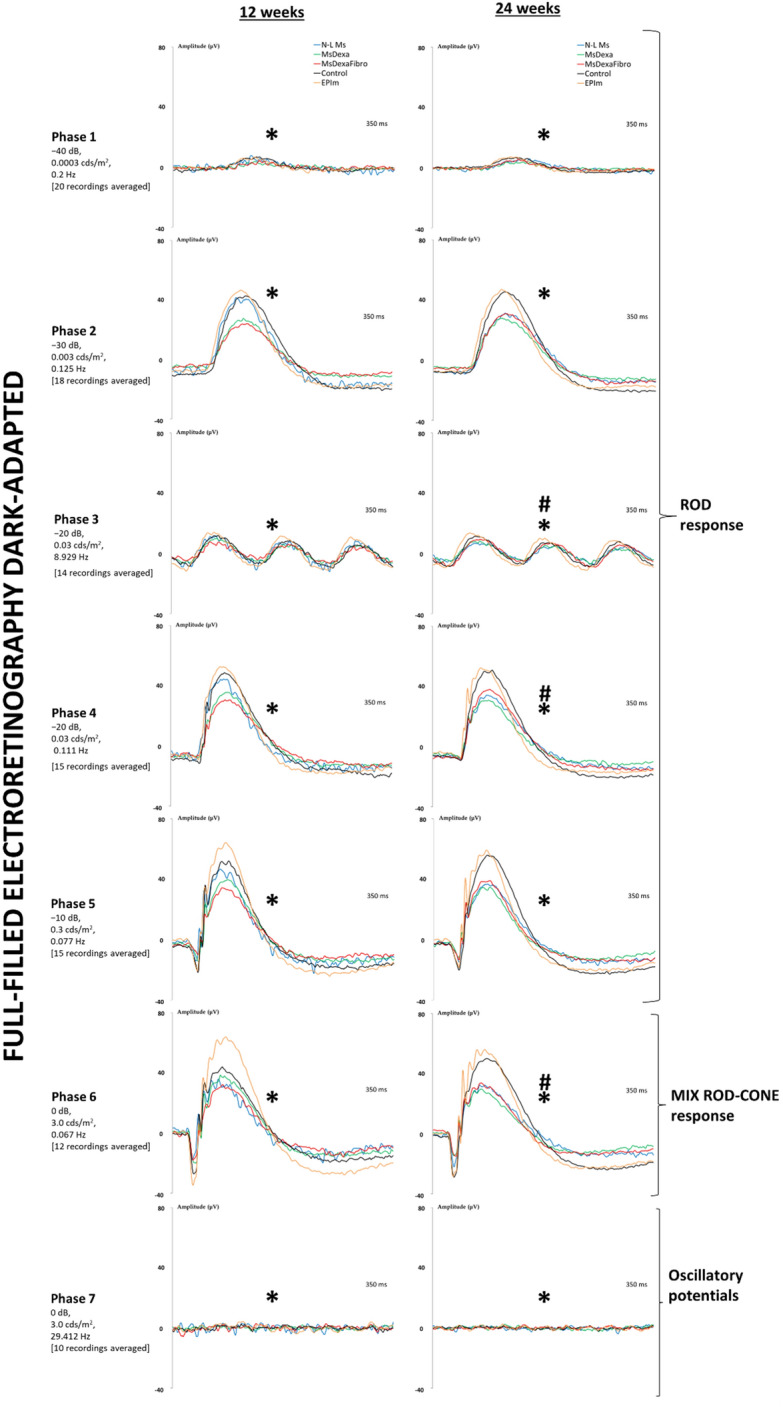
Follow-up comparison of neuroretinal function using dark-adapted electroretinography between four chronic glaucoma models and healthy control cohorts. Full-field ERG test. *μV* microvolts; *ms* milliseconds; *dB* decibel; cds: candels; *m*^*2*^ square meter; *Hz* hertzio; *N-L Ms* cohort with non-loaded microspheres; *MsDexa* cohort with microspheres loaded with dexamethasone; *MsDexaFibro* cohort with microspheres loaded with dexamethasone and fibronectin; *EPIm* cohort with sclerosis of the episcleral veins;*: statistical significance (*p* < 0.05) between glaucoma models and healthy controls (ANOVA); #: statistical significance between the episcleral vein sclerosis model and each microsphere model by Scheffé test.

**Figure 9:**
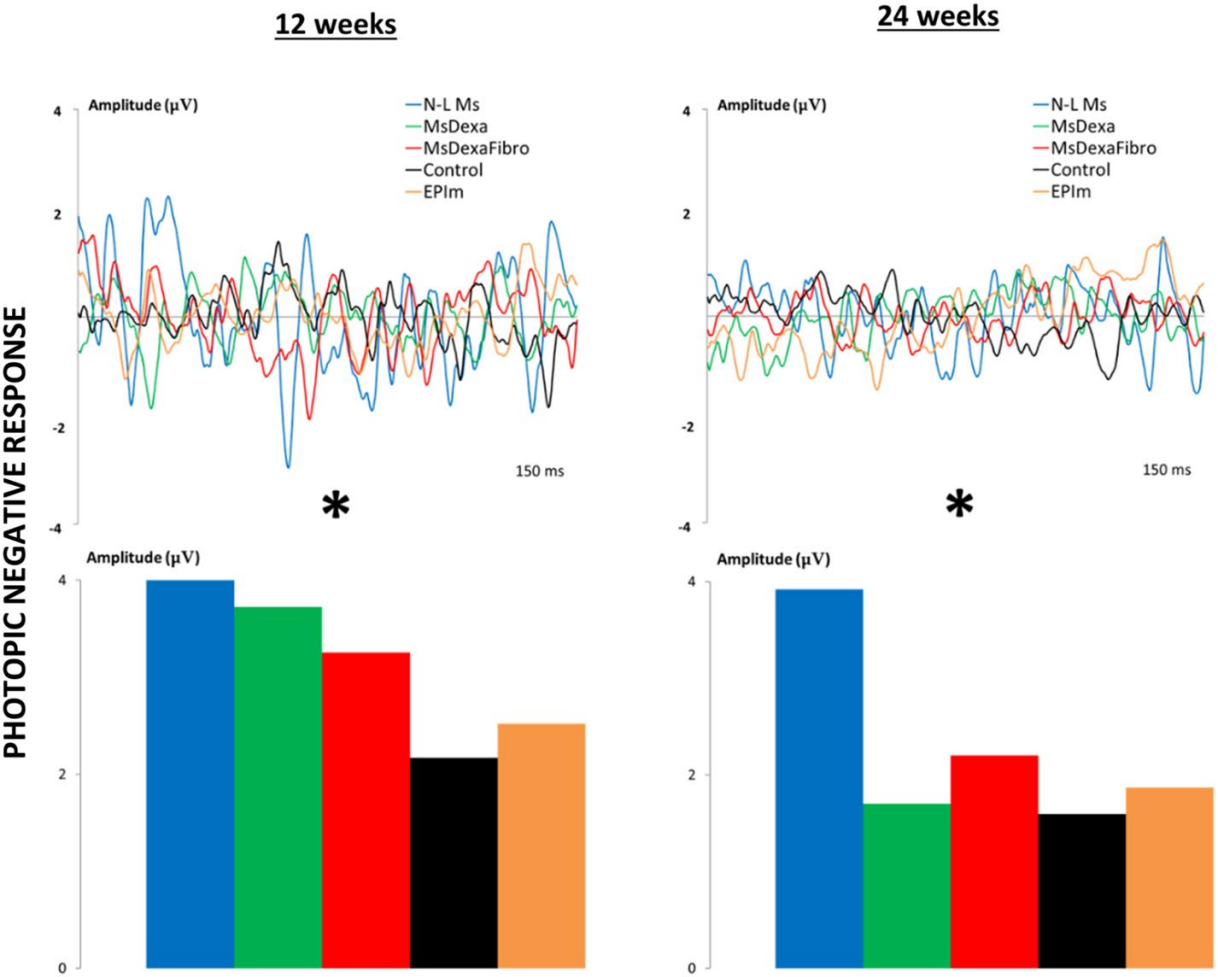
Follow-up comparison of neuroretinal function using light-adapted electroretinography between four chronic glaucoma models and healthy control cohorts. Photopic negative response test. Retinal ganglion cell functionality increased in all glaucoma models at middle times explored. *N-L Ms* cohort with non-loaded microspheres; *MsDexa* cohort with microspheres loaded with dexamethasone; *MsDexaFibro* cohort with microspheres loaded with dexamethasone and fibronectin; *EPIm* cohort with sclerosis of the episcleral veins. *: statistical significance (*p* < 0.05) between glaucoma models and healthy controls (ANOVA).

The scotopic ERG showed statistically different decreasing signals among cohorts at weeks 12 and 24. The MsDexa and MsDexaFibro cohorts presented the earliest decrease in outer retinal function. However, increased signal—even higher than in the healthy control cohort—was observed in the EPIm cohort (Fig. 8). According to the PhNR protocol that explores ganglion cell function, although a decrease in signal was observed over the course of the study in all cohorts, the four induced chronic glaucoma cohorts presented higher measurements than the healthy control cohort. In this test, the N-L Ms cohort presented the highest signal, the EPIm cohort the lowest signal in the early phases, and the MsDexa cohort the lowest signal in the later phases (Fig. 9).

## Discussion

Glaucoma animal models previously created by cauterization or sclerosis of episcleral veins and blockage of trabecular meshwork with ferromagnetic microspheres, or combined with tamponade substances such as hyaluronic acid, often produce intraocular pressure increases characterized by abruptly elevated levels (approximately 30 mmHg) in the early stages that then progressively decline^9^. In contrast, the intraocular pressure increase in patients with primary open angle glaucoma is gradual. The above models are therefore far from reproducing the normal clinical course of the disease. However, models developed with biodegradable microspheres produce a slower hypertensive curve that emulates primary open angle glaucoma in humans. In this paper, we compared the episcleral vein sclerosis model (EPIm) with three different chronic glaucoma models induced by intracameral injection of biodegradable PLGA microspheres (non-loaded^14^, loaded with dexamethasone^15^, and loaded with dexamethasone and fibronectin^16^). Using PLGA offers several advantages: on the one hand, it permits creation of microsystems capable of microencapsulating one or more active agents to subsequently release them progressively over several months^32^, allowing the constant and sustained presence over time of the chosen harmful compounds in the trabecular meshwork; on the other, this alteration of the trabecular meshwork is achieved without damaging any other ocular structure, since PLGA is a highly biocompatible biomaterial that hydrolyzes into its monomers, which subsequently disappear without trace in the Kreb cycle. In fact, PLGA is authorized for use at intraocular level^33^.

Overall, under the above conditions EPIm caused the sharpest intraocular pressure increase with the highest initial vitreous signal and the greatest final GCL loss, while non-loaded microspheres injection produced a more progressive intraocular pressure increase and somewhat milder neuroretinal damage. However, even though ocular hypertension was not reached, the model with non-loaded microspheres generated the second-highest vitreous intensity and repeated injections increased the vitreous signal by the end of the time period, suggesting an inflammatory response secondary to intraocular injection that influences glaucoma^34^. MsDexa produced moderately elevated and sustained intraocular pressure levels (highest cumulative intraocular pressure) that generated the greatest RNFL loss; and finally, MsDexaFibro produced the greatest fluctuations in intraocular pressure, neuroretinal OCT and the vitreous. The steroid induced glaucoma model (with the potential anti-inflammatory effect of dexamethasone and induced with fewer intraocular injections) presented lower sustained vitreous parainflammation over time. This lower vitreous parainflammation was not accompanied, however, by neuroretinal protection, in fact the opposite occurred, as there was worse functionality and a higher percentage neuroretinal loss at the end of the study. This simulates the steroid glaucoma that occurs in humans. The different induced models presented specific characteristics that make it possible to compare the different risk factors influencing multifactorial glaucoma, such as (1) the acute, steady, progressive and fluctuating increase in intraocular pressure, (2) the parainflammation detected in the vitreous and the effect on it of the models induced by corticosteroid anti-inflammatory drugs, and (3) the induction method itself.

Axonal degeneration correlates strongly with intraocular pressure^3^. In this respect, MsDexa—with the highest intraocular pressure levels—presented the highest percentage of RNFL loss. It was followed in second place by RNFL loss in the MsDexaFibro cohort, which exhibited greater fluctuations. EPIm—with the highest glial response^14^ and elevated intraocular pressure—exhibited the highest percentage of GCL loss in OCT. These results support previous evidence that elevated intraocular pressure is the main risk factor affecting axons, while other factors such as fluctuations^4^ or increased glial activation and immune response cause progression and further secondary neurodegenerative damage^35^.

As in previous papers, in the models presented in this study the greatest loss of retinal ganglion cell, quantified as a decrease in GCL thickness, was detected in the internal sectors of the horizontal axis, where it followed a temporal > superior > nasal > inferior sectorial loss trend aligned with the regions with highest densities. On the vertical axis, a superior > inferior > nasal > temporal loss trend was detected in the RNFL sectors, as observed by OCT^18,19,24,36^. In addition, retinal neurodegeneration produces an increase in thickness due to the increase in cell size and the increase in immune response^37,38^. In our study, the greatest increase in thickness occurred in the MsDexaFibro model—with immunogenic potential^39,40^—which by the end of the study presented the greatest percentage loss of retinal posterior pole thickness and the least outer and intermediate retinal layer function. In addition, during secondary neurodegeneration retinal ganglion cell loss have been described to occur in clusters^41^. To get this result, previous authors have relied on models reaching intraocular pressure levels above 30 mmHg (which can create alterations in vascularization similar to branch venous occlusion^10^), employing shorter periods and histological studies, which makes it impossible to perform serial scans on the same animal^42,43^. In our study comprising 6-month chronic glaucoma models and non-invasive OCT scans, neuroretinal loss was analyzed topographically, showing ordered patterns of secondary retrograde degeneration, without the need to euthanize animals. Recently, similar contiguity spread damage was demonstrated in another optic neuropathy, using computational mathematical modeling^44^. Furthermore, it also allowed us to study the relationship with retinal function, in which dysfunction appears to occur prior to cell loss. Interestingly, in all the chronic glaucoma animal models presented in this study, we found increased retinal ganglion cell function with the PhNR test and increased function of the outer layers in the episcleral model with the scotopic ERG versus controls. It seems that supranormal responses may be suggestive of pathology^45^. In loop-induced^46,47^ and microspheres-induced ocular hypertensive models^48^, an increase in ERG signal has been described as a reflection of synaptic imbalance at the onset of intraocular pressure increase. And a recent study demonstrated a structural plasticity with increased synaptic connectivity with rods while undergoing glaucoma degeneration^49^. This hyperactivity may be one of the earliest dysfunctions of the pathophysiological process of neurodegeneration^50^ (suggested in other neurodegenerative diseases such as Alzheimer’s disease^51^ or multiple sclerosis^52^). Our results detected hypersignals of retinal ganglion cell function even with mild intraocular pressure increases (21 mmHg), and outer layers with elevated intraocular pressures (approximately 30 mmHg) in early stages (12 weeks) that then decreased in later stages. This corroborates an increased retinal ganglion cell susceptibility to glaucomatous damage, but also an involvement of the outer layers^53,54^.

OCT analysis of the vitreous allows in-depth study of the vitreous parainflammation caused by different models of ocular hypertension^22^. Under normal conditions, the vitreous is transparent. Therefore, the activation of hyperreflective vitreous opacities could be due to immune cells produced as a consequence of induced glaucoma. As can be seen in Fig. 7, this parainflammation can be detected and quantified by non-invasive OCT imaging using vitreous/retinal pigmented epithelium relative intensity, total area and number of hyperreflective opacities. Thus, these parameters could serve as highly useful biomarkers of chronic glaucoma. The influence of immunity on glaucoma has been evidenced in histological studies in neuroretina^55^. This study demonstrates the monitoring of immunity in vitreous and without the need to euthanize animals.

In conclusion, biodegradable non-loaded microspheres models and microspheres models loaded with corticoid or a mixture of corticoid and fibronectin allow development of different models of chronic glaucoma with specific and individual characteristics, thus identifying and analyzing different factors involved in the onset and progression of glaucoma. These models help to increase knowledge of the physiopathology mechanism of the disease, thereby steering treatment towards precision medicine. Considering their chronicity, the models presented in this paper can be considered valuable tools for evaluating the efficacy of neuroprotective drug delivery systems.

## Data Availability

The datasets are available upon reasonable request from the corresponding author.
